# Dyspepsia

**Published:** 1874-11

**Authors:** 


*NEW YORK MEDICAL RECORD—September*, 1874.

Dyspepsia.—Dr. Leven (*Lyon Medical*, No. 7, 1874,) read recently in the Academy of Medicine a paper on the physiological pathology of the stomach, and particularly concerning dyspepsia. According to Leven, the stomach has only the secondary part to play in digestion—it reduces, by means of its movements, and the juices secreted, the volume of the aliments, breaks them up, and facilitates their entry into the intestines; thus, its function is that of the triturating machine.

The injection of nitrogeneous substances does not augment the secretion of gastric juice, but produces a great amount of watery fluid due to exosmosis from the capillaries of the mucous membrane. According to this author, dyspepsia is an affection of the stomach characterized by an aqueous secretion, which retards digestion and injures it. The hypersecretion is in some way commenced by the organ to get rid of foreign bodies, over which it has little power and which are expelled in a quantity of fluid. Perhaps in all cases of dyspepsia the predominant symptom is hypersecretion of fluid; and treatment should be directed against this. But the classic remedies, such as quinine, carbon, pepsin, etc., are usually inefficacious. Doubtless these often give relief, but rarely cure. For this reason Dr. Leven has abandoned these and makes use of the following treatment, which in many cases has cured even the most inveterate dyspepsia. He advises his patients to take small doses of neutral salts, or others attacked with difficulty by the stomach, sulphate of soda, phosphate of soda, phosphate of lime, chloride of sodium, or bromide of potassium. He never gives doses larger than from four to eight grains in the day of such remedies, and soon notices an improvement in the symptoms of dyspepsia; the swelling of the stomach diminishes, the secretion is less exaggerated, and the disease is gradually cured. Besides this, a diet easily digested is recommended.

NOTE.—The use of the hypo-sulphite of sodium, in doses of ten grains before meals, is found to be very efficacious in the forms of dyspepsia mentioned above.—EDITOR.

